# Pseudophakic angle-closure from a Soemmering ring

**DOI:** 10.1186/s12886-016-0257-6

**Published:** 2016-06-17

**Authors:** Yanin Suwan, Bayasgalan Purevdorj, Chaiwat Teekhasaenee, Wasu Supakontanasan, Pornchai Simaroj

**Affiliations:** Department of Ophthalmology, Faculty of Medicine, Ramathibodi Hospital, Mahidol University, Bangkok, Thailand

**Keywords:** Soemmering ring, Angle-closure, Pseudophakic angle-closure, Cataract, Ultrasound biomicroscopy

## Abstract

**Background:**

Report of three patients with pseudophakic angle-closure from a Soemmering ring. Three mechanisms of the Soemmering ring induced pseudophakic angle-closure in three patients were demonstrated by meticulous anterior segment examination and ultrasound biomicroscopic (UBM) analysis.

**Case presentation:**

In the first case, the Soemmering-capsule-IOL complex caused relative pupillary block similar to a phakic eye and was successfully treated with laser iridotomy alone. In the second case, an enlarged Soemmering ring provided posterior iris support in apposition to the anterior chamber angle. We performed a laser capsulotomy through the iridotomised hole. The last, a protruding Soemmering content causing absolute pupillary block became resolved after laser iridotomy and total Soemmering ring content removal.

**Conclusion:**

Angle-closure in pseudophakic eyes is uncommon. Several causes have been reported in the literatures including Soemmering ring. This is the first report on three different mechanisms of Soemmering ring related angle-closure in pseudophakic eyes. Ultrasound biomicroscopic analysis plays a crucial role as a diagnostic tool.

## Background

Secondary angle closure in pseudophakic eyes is infrequent. The pressure rise may be temporary or permanent, depending on the underlying mechanism. Pupillary block is the most common cause of angle closure following cataract surgery and intraocular lens (IOLs) implantation [1, 2]. The pupillary aperture can become occluded by any surface that it comes into apposition with, whether this surface is behind or in front of it [1]. The causes of pupillary block after cataract surgery include postoperative iridocyclitis with seclusio pupillae, dense and impermeable anterior hyaloid membrane (malignant glaucoma), adhesion between the pupil and IOL (pupillary block glaucoma), adhesion among the capsule-IOLs-iris complex (posterior pupillary block glaucoma), pupillary block by air or silicone, inadequate iris openings, swollen lens material behind the iris, and free vitreous block [1, 3].

One of the rare causes of angle closure in a pseudophakic eye is the proliferation of the remaining lenticular epithelial cells in the peripheral part of the capsular bag resulting in a thick circumferential structure at the level of the lens called “Soemmering ring.” It sometimes develops into having the appearance of “a string of sausages” or “donut shape.” This rare benign structure has been reported to cause pupillary block, leading to angle closure. Pathologically, the ring of Soemmering may briefly be defined as the capsular remains of some retained lens epithelial cells, the center of the lens having been absorbed after either operative or traumatic penetration [4]. The ring can readily be detected but more clearly in some cases than in others, in the average aphakic eye in which the lens has not been removed in its capsule. Subclinical anterior chamber inflammation associated with exfoliation syndrome may have contributed to the excessive growth of the Soemmering ring and the progression of synechial angle closure [5].

The first patient reported here had acute angle closure with pupillary block, due to an enlarging Soemmering ring after phacoemulsification, while the second patient had chronic progressive synechial angle closure without pupillary block. The third case had acute angle closure glaucoma from enlarged Elschnig pearl. We are reporting three different mechanisms of Soemmering ring induced secondary angle closure in pseudophakic patients.

### Case 1

**Fig. 1:**
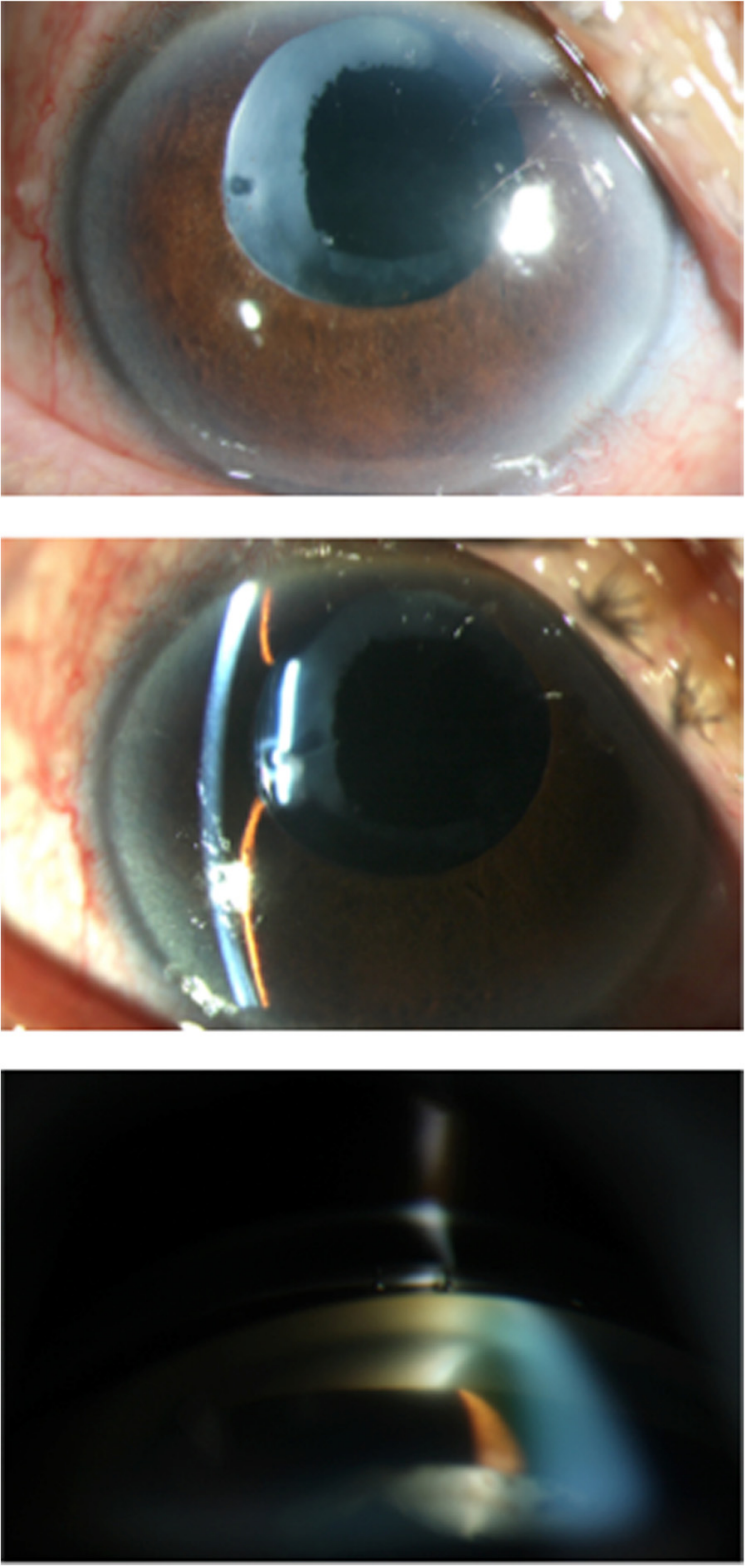
Slit-lamp photographs of anterior segment before neodymium: YAG laser peripheral iridotomy. **a**, **b** Pseudophakic eye with “donut” like circumferential capsular opacity and anterior chamber is centrally deep and peripherally shallow. **c** Gonioscopic examination showed whitish mass (*arrow*) or Soemmering ring between iris and IOL

An 74-year-old Thai male had intermittent blurred vision, red eye and pain in the right eye for 5 days. He had a history of phacoemulsification with a posterior chamber intraocular lens in the right eye 25 years previously. Initial slit-lamp biomicroscopy in the right eye revealed elevated intraocular pressure (IOP) with posterior capsular opacification. He was treated by Nd:YAG laser capsulotomy and topical anti-glaucoma medications (Bimatoprost Ophthalmic Solution 0.03 %; Brimonidine tartrate 0.15 %; Timolol maleate 0.5 %). However, the patient’s symptoms were not improved and he was referred to our center. Best corrected visual acuity was 20/200 OD and 20/100 OS. Slit lamp biomicroscopy in the right eye revealed corneal edema, iris bombé without detectable posterior synechaie. Slit-lamp biomicroscopy in the left eye was unremarkable. His IOP was 46 mmHg OD and 12 mmHg OS. Gonioscopy revealed a convex iris with 360° peripheral anterior synechiae prominent Soemmering ring behind the iris OD and widely opened angle OS (Fig. 1a–c).

A-scan ultrasound biometry showed shallow central anterior chamber depth (ACD) in the right pseudophakic eye (Axial length (AXL) = 26.81 mm; IOL; Anterior chamber depth (ACD) =1.87 mm), and normal central anterior chamber depth in the left eye (AXL = 26.68 mm; Lens thickness (L) = 4.66 mm; ACD = 3.35 mm). Condensing lens ophthalmoscopy revealed tigroid fundus with full cupped optic disc OD and cup to disc ratio 0.4 OS.

**Fig. 2:**
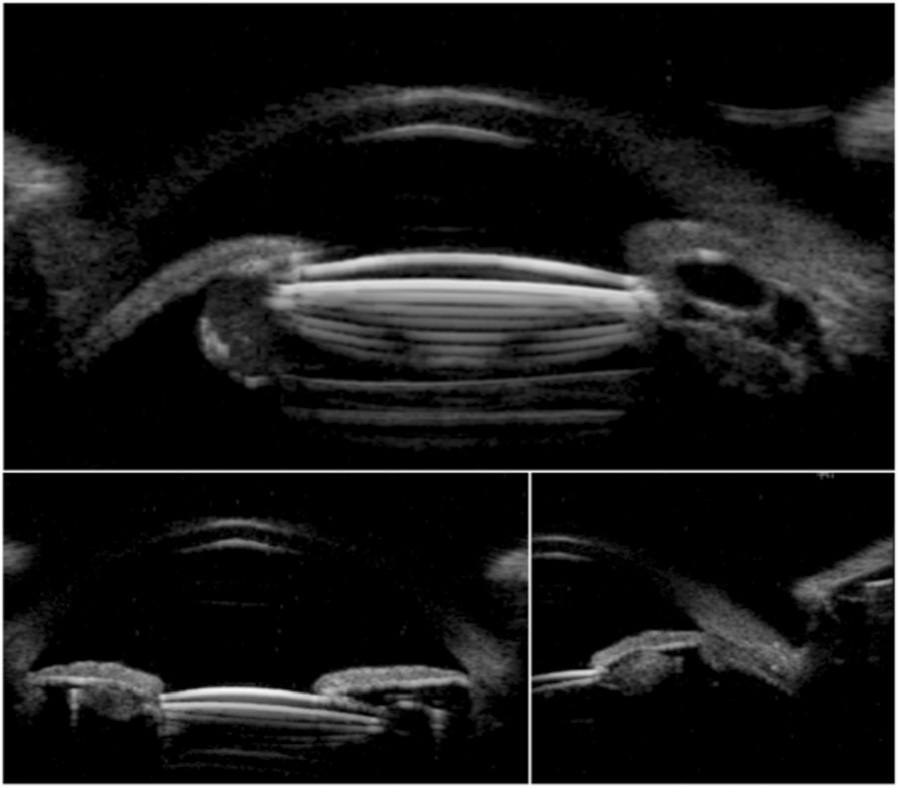
Ultrasound biomicroscopic images before laser treatment. (**a**) The ovoid structure beyond optic edge was shown with iris bombe. (**b**) After laser treatment, resolution of pupillary block with deepening of anterior chamber. IOL was decentered. (**c**) Anterior chamber angle was opened wide

B-scan ultrasound biomicroscopy demonstrated a large circumferential structure behind the posterior iris surface with pupillary block (Fig. 2a).

**Fig. 3:**
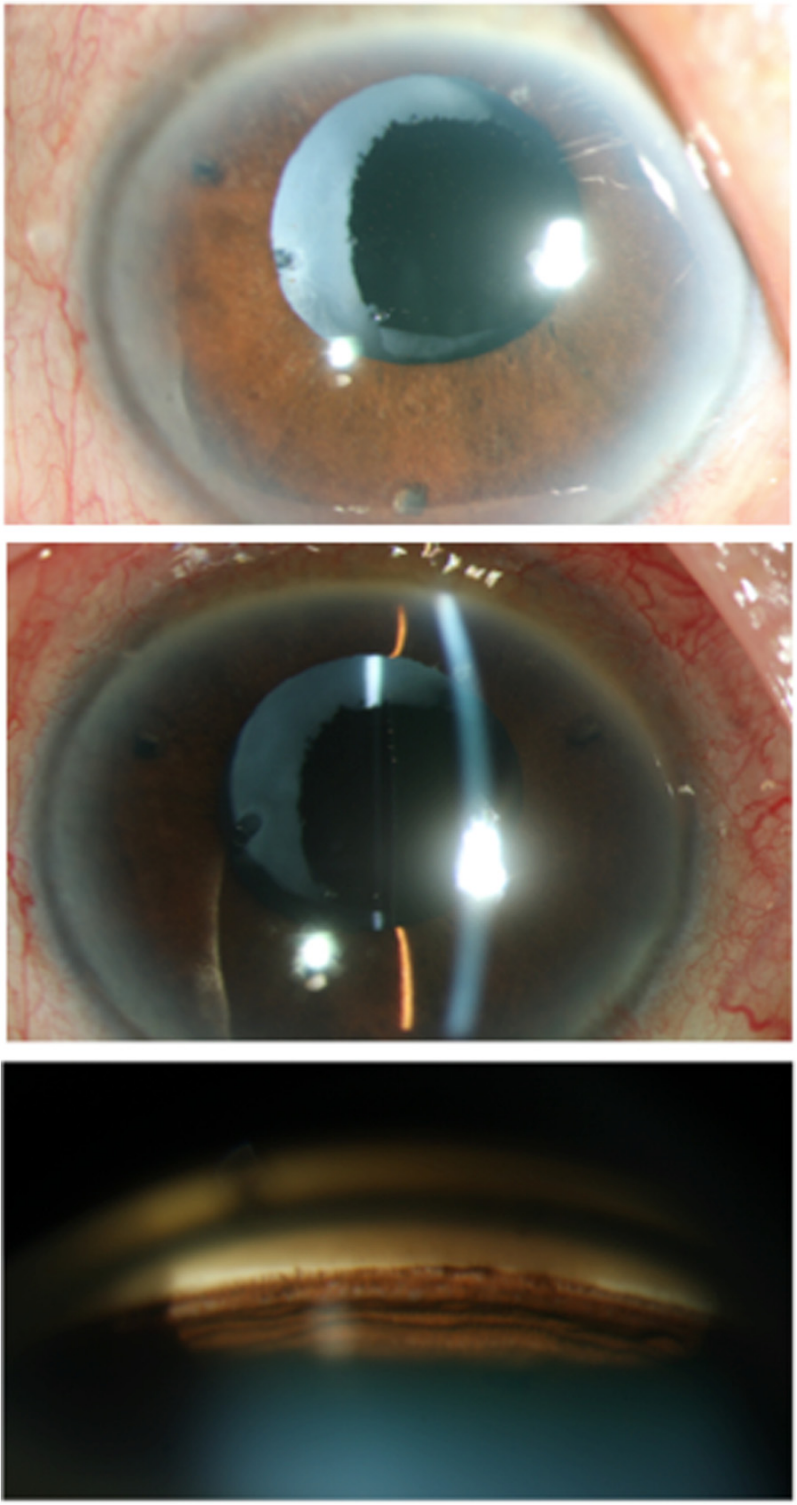
Slit-lamp photographs of anterior segment. Nd: YAG laser iridotomy was performed at 2, 6 and 10 o’clock. **a**, **b** Immediately after laser surgery the central anterior chamber was deepened. **c** Anterior chamber angle was opened

Therapeutic laser iridotomies (LPI) without capsular penetration was performed at 2, 6 and 10 o’clock OD in order to avoid aqueous loculation from undetectable septums between posterior iris surface and anterior portion of Soemmering ring. Immediately repeated gonioscopy showed an opened anterior chamber angle more than 210° (Fig. 3a–c). Repeated A-scan ultrasound biometric measurements showed a deepened ACD OD (AXL = 26.89 mm; IOL; Vitreous length (V) = 22.28 mm; ACD = 3.25 mm). Postoperative slit-lamp biomicroscopy showed marked pseudophacodonesis. Post LPI IOP was decreased to 10 mmHg with deepened anterior chamber (AXL = 26.58 mm; L = 0.62 mm; V = 21.83 mm; ACD = 3.68 mm) and gonioscopy revealed resolution of pupillary block and an opened anterior chamber angle more than 270°. IOP could be controlled with two topical antiglaucoma medications.

### Case 2

**Fig. 4:**
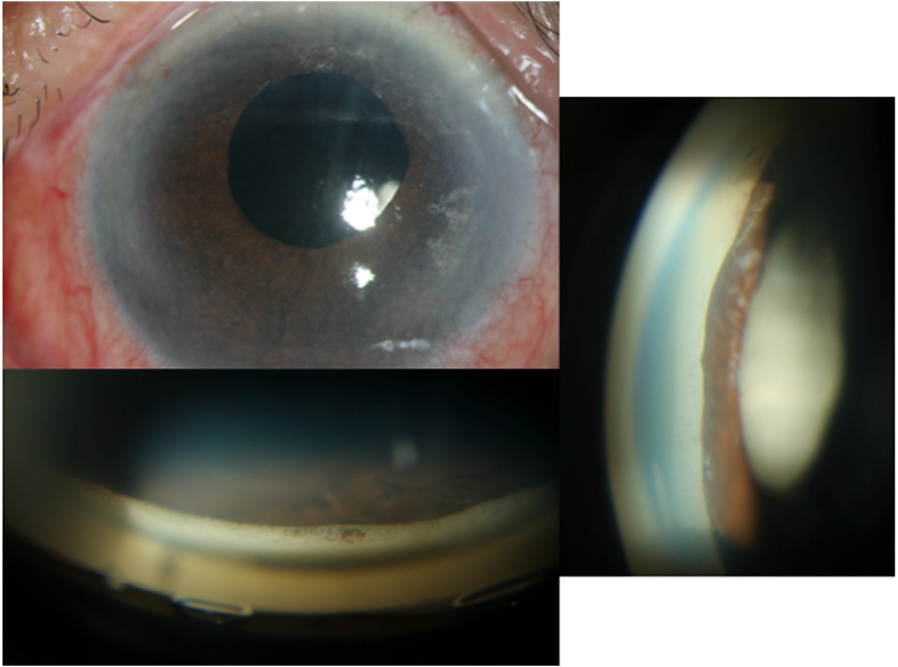
Before laser treatment. **a** Shallow both central and peripheral anterior chamber. **b**, **c** Gonioscopy shows peripheral anterior synechiae. The Soemmering ring is shown as a whitish ellipsoid structure behind the iris. The Soemmering ring pushes the iris behind and the angle closure occurs

**Fig. 5:**
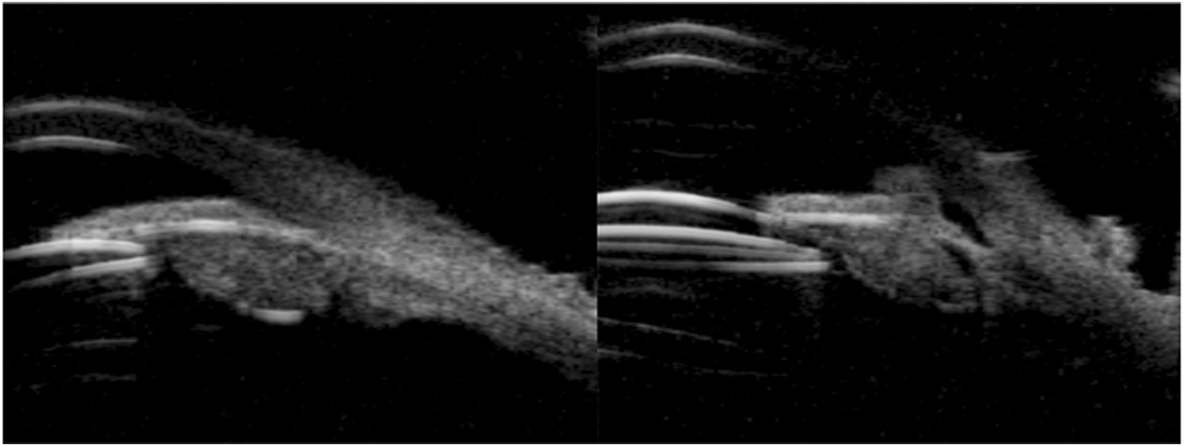
Ultrasound biomicroscopic images before laser treatment (**a**) Ovoid hyperechoic structure supported posterior iris surface without pupillary block. **b** After laser treatment, hyperechoic content moving into anterior chamber through iridotomised hole with deepening of anterior chamber

**Fig. 6:**
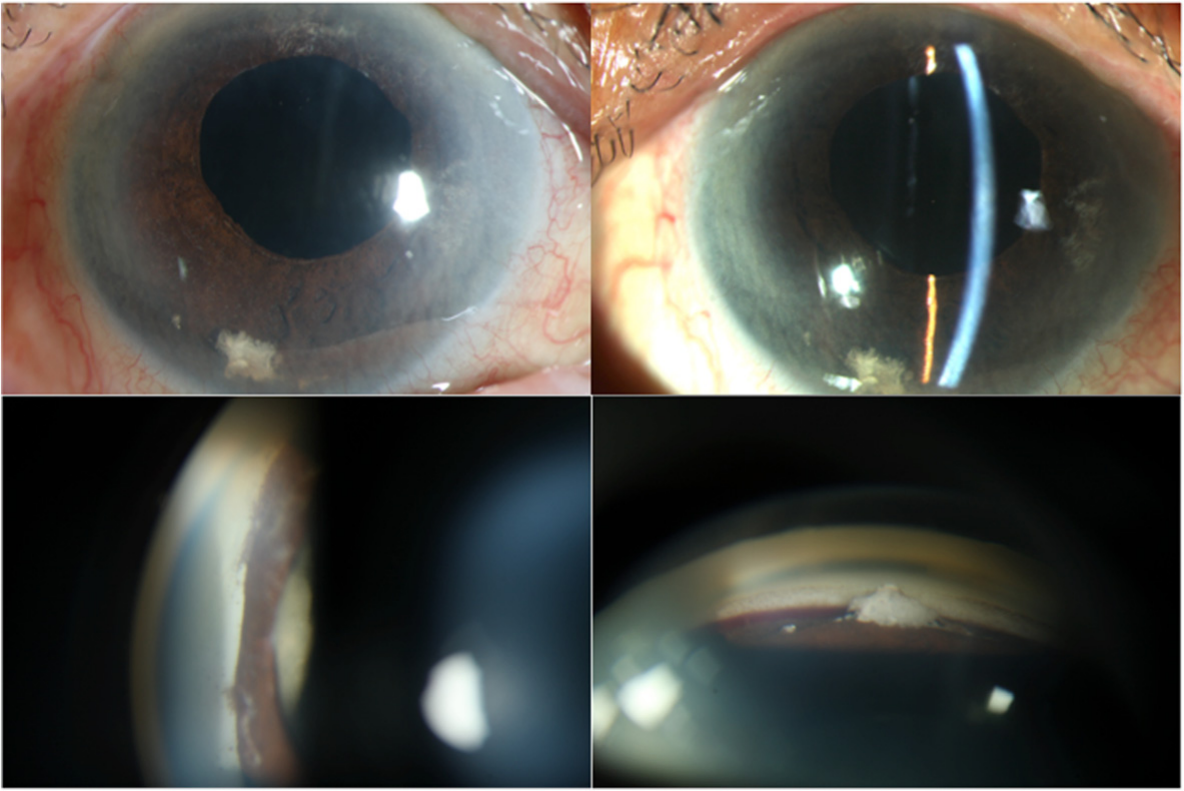
Immediately after laser treatment. **a**, **b** Deepened anterior chamber in center and periphery as well. **c**, **d** Soemmering ring was significantly reduced in size and the angle was opened. Gonioscopy showed an opened angle and particles of Soemmering ring in anterior chamber penetrated through the hole of laser iridotomy

An 81-year-old Thai female presented with a painless visual loss in the right eye. She had a history of phacoemulsification with intra-ocular lenses implantation of both eyes and Nd:YAG laser capsulotomy after the surgery. The patient was treated for chronic glaucoma and was taking latanoprost 0.005 % and timolol maleate 0.5 % OD. Best corrected visual acuity was to count fingers at 3 ft OD and 20/40 OS. IOP was 32 mmHg OD and 6 mmHg OS. Slit lamp biomicroscopy revealed shallow anterior chamber OD, while OS was unremarkable. A-scan ultrasound biometric measurement showed ACD was 1.61 mm OD and 3.79 mm OS. Gonioscopy revealed 360 degree-peripheral anterior synechiae OD and normal opened angle OS (Fig. 4a–c). Ultrasound biomicrosopy revealed a large hyperechoic structure in apposition to the posterior iris surface (Fig. 5). Therapeutic laser iridotomy was performed in the 6 o’clock positions which is the most protruded area. We found the anterior chamber was deepened immediately. Subsequent gonioscopy found opened anterior chamber angle more than 210° and particles of a Soemmering ring, appearing as part of a secondary cataract, moving into the anterior chamber through the iridotomised hole (Fig. 6a–c). A-scan ultrasound biometric measurements showed the anterior chamber was deepened (ACD = 3.25 mm) OD. At last follow up, her IOP was controlled by two topical ocular hypotensive medications.

### Case 3

**Fig. 7:**
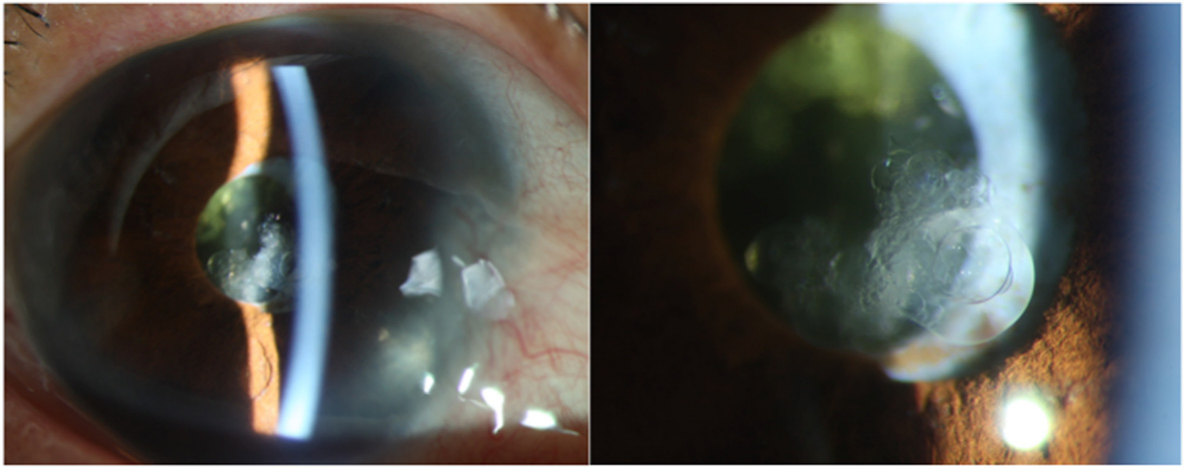
Slit-lamp photographs of anterior segment before laser treatment. (**a**) Deep central and shallow peripheral anterior chamber with Elschnig pearl protruding through the pupillary aperture. (**b**) The close-up images revealed an enlarged Elsching’s pearl in a multiloculated nature

A 64-year-old Thai female presented with a painful visual loss in the right eye for 1 week. She had a history of acute angle closure glaucoma OD and also had a history of phacoemulsification, intraocular lens implantation, and goniosynechialysis 5 years previously. Her visual acuity was 20/40 OD and 20/20 OS, IOP 30 mmHg OD 12 mmg OS. Slit-lamp biomicroscopy revealed protruding Soemmering content via pupillary aperture into anterior chamber (Fig. 7a–b). Protruded Soemmering content led to pupillary block which had been relieved by therapeutic laser iridotomy. Post-operative gonioscopy showed an opened anterior chamber angle. Residual Soemmering content was removed by anterior chamber aspiration. Her IOP was under the normal range without antiglaucoma medication.

## Conclusion

Angle closure in pseudophakic eye is uncommon. From the classification of angle closure at 4 anatomical levels (iris, ciliary body, lens and posterior to the lens), pupillary block is the most common cause of angle closure in pseudophakic eye [2, 6]. We are reporting different mechanisms of Soemmering ring related angle closure.

In the first case, the Soemmering-capsule-IOL complex caused relative pupillary block similar to phakic eye due to increased thickness in the equatorial zone. Iris bombé without detectable posterior synechiae were found compatible with anterior pupillary block diagnosis. After a successful laser iridotomy without capsular penetration, we observed that the gush of aqueous had passed through the iridotomised hole and suddenly deepened the anterior chamber. Generally, after resolution of anterior pupillary block, the intraocular lens should be in its normal position without backward movement. Nevertheless, we observed backward movement of the intraocular lens with deepening of the anterior chamber due to pseudophacodonesis related zonular insufficiency.

In the second case, the enlarged Soemmering ring provide posterior iris support in apposition to the anterior chamber angle. We performed capsulotomy through the iridotomised hole and found after photodisruption of the capsule and Soemmering ring, the anterior chamber deepened and the iridocorneal touch resolved with part of the Soemmering ring content moving into the anterior chamber through the iridotomised hole. This result is similar to previous reports that many aphakic and pseudophakic eyes without iridectomy do not develop pupillary block, whereas others still do despite patent iridectomy [2, 3].

In the third case, the protruding Soemmering content caused absolute pupillary block in which the ring itself acts as a one way valve to prevent anterior-posterior chamber pressure balance. Laser iridotomy equalized the two chambers’ pressure. Total Soemmering ring content removal prevents further egress of Elsching’s pearl into the anterior chamber.

In patients with Soemmering ring related angle closure, evaluation of posterior chamber structure behind the iris is important. Ultrasound biomicroscopy plays a crucial role in demonstrating the anatomical relationship between angle-IOLs-Soemmering-ciliary complex. In three patients, we did not found any anterior ciliary body positioning which can further aggravate angle-closure in previous report [7]. Multiple iridotomy/capsulotomy may be required because of the loculated nature of Soemmering ring. Intensive anti-inflammation is an obligatory post laser treatment to prevent re-closure of the newly opened anterior chamber angle. The cortical clean up after nuclear removal is also an imperative surgical step in a cataract surgery to prevent Soemmering formation. Some studies recommended if there is a large Soemmering ring present after previous cataract surgery, the technique involves opening the bag over the ring with a new anterior capsulotomy and removing the secondary cataract in the Soemmering ring, creating a peripheral capsular bag in which the haptics can be placed for fixation of the existing or a secondary IOLs [8].

## Abbreviations

ACD, anterior chamber depth; AXL, axial length; L, lens thickness; LPI, laser peripheral iridotomy; IOL, intraocular lens; IOP, intraocular pressure; Nd:YAG, neodynium – doped yttrium aluminium garnet; UBM, ultrasound biomicroscopy; V, vitreous length
